# Aircraft observations in a tropical supercluster over the equatorial Indian Ocean during MISO-BOB field campaign

**DOI:** 10.1038/s41598-024-51527-4

**Published:** 2024-01-25

**Authors:** Jayesh Phadtare, Harindra J. S. Fernando, Garrett Black, Kaitlyn McLaughlin, Jeremy Dehart, Raghavendra Krishnamurthy, G. S. Bhat, Emily Shroyer, Amit Tandon, Jaynise M. Pérez Valentín, S. U. P. Jinadasa

**Affiliations:** 1 Department of Civil and Environmental Engineering and Earth Sciences, University of Notre Dame, Notre Dame, IN 46556 USA; 2 Department of Aerospace and Mechanical Engineering, University of Notre Dame, Notre Dame, IN 46556 USA; 3 53rd Weather Reconnaissance Squadron, Keesler Air Force Base, Biloxi, MS 39534 USA; 4 Pacific Northwest National Laboratory, Richland, WA 99352 USA; 5 Centre for Atmospheric and Oceanic Sciences, Indian Institute of Science, Bengaluru, 560012 India; 6 College of Earth, Ocean, and Atmospheric Sciences, Oregon State University, Corvallis, OR 97331 USA; 7 Department of Mechanical Engineering, University of Massachusetts Dartmouth, North Dartmouth, MA 02744 USA; 8 Faculty of Engineering and Management, Ocean University of Sri Lanka, Colombo, 15 Sri Lanka; 9 Present Address: Office of Naval Research, Arlington, VA 22203 USA

**Keywords:** Atmospheric dynamics, Physical oceanography

## Abstract

The Monsoon Intra-Seasonal Oscillations in the Bay of Bengal (MISO-BOB) field campaign was conducted in the Indian Ocean during the 2018 and 2019 summer monsoon seasons. WC-130J aircraft of the 53rd Weather Reconnaissance Squadron of the US Air Force participated in the campaign in June 2018. The dropsonde observations across a tropical supercluster showed zonal wind variations in association with the structure of the convectively coupled Kelvin wave (CCKW). Within the supercluster, easterlies (westerlies) were observed in the upper (lower) troposphere; this transformation occurred just below the 0^∘^ C level. The cold pool had an easterly component throughout, and it was coldest (by 2.5^∘^ C) at the center of the supercluster, deepest ($\sim$ $1000\,\hbox {m}$) at its rear/western end, and shallowest ($\sim$ 300 m) at the front/eastern end. The level of free convection (LFC) at the front end was at $897\,\hbox {m}$ altitude. At the eastern flank of the supercluster, zonal convergence in the lower troposphere occurred between 500-1500 m levels above the surface between the westerlies within the supercluster and opposing ambient easterlies. Thus, the uplifting of conditionally unstable air parcels above LFC to the east of the supercluster was likely to occur due to this convergence rather than the cold pool influence. Conversely, the western flank of the supercluster had low-level zonal divergence. These observations support the notion of ‘self-similarity’ among the mesoscale convective systems and large-scale waves.

## Introduction

Accurate forecast of South Asian monsoon is challenging, but it is a crucial task as the seasonal rainfall of monsoon provides at least 80% of fresh water to about 2 billion inhabitants of this region. Further, monsoonal winds, rainfall and other associated parameters exhibit a prominent variability on intraseasonal timescales^1^ and understanding the physical processes in the atmosphere and ocean over the equatorial Indian Ocean (EIO) during different phases of intraseasonal oscillation (ISO) is vital for monsoon forecasting. Despite the advancements of satellite and computing technologies, present-day global circulation models perform poorly in long-range and seasonal forecasting of monsoon; imperfect parameterization of subgrid processes appear to be a primary culprit^2–4^. Further improvement in monsoon forecasting, therefore, requires a better understanding of subgrid processes and improving their parametric representation in models, often referred to as ‘model physics’. Monsoonal rainfall is a consequence of organized convection^5–10^ with moisture source being the warm tropical ocean^11^. Thus, improvements in the modelling of air-sea interaction^12–17^ and convection^4,18–21^ may result in substantial reduction of forecast errors. This calls for high-resolution in situ observations of the tropical atmosphere-ocean coupled system. The paucity of pertinent high-quality observations motivated the ‘Monsoon Intraseasonal Oscillations in the Bay of Bengal’ (MISO-BOB) research program^22,23^.

Unlike in the equatorial Western Pacific, where observations from several weather stations over the maritime continent have provided a vast amount of meteorological data^24^, the EIO has fewer island weather stations. A prominent mode of tropical ISO, namely, the Madden-Julian Oscillation (MJO), originates and matures over the EIO while propagating eastward^25^. During the Boreal summer, the eastward-moving MJO may trigger a northward-moving cloud band in the southern Bay of Bengal, heralding a transition from the break to the active phase of monsoon over the Bay of Bengal and the Indian subcontinent^26–28^. Some researchers refer to the summer-time ISO as the Boreal Summer Intraseasonal Oscillation (BSISO)^29^. This term encompasses the northward and eastward movement of ISO over the EIO. Ship and aircraft observations of ISO events over the EIO exist but are inadequate^22,30–32^. Ships move relatively slowly and provide an almost continuous time series of the desired variables along their tracks, however, these observations are unsuitable for obtaining the spatial structure of convective systems. Conversely, aircraft is capable of taking vertical and horizontal sections across a convective system nearly simultaneously using dropsondes and onboard instruments^33–39^.

The MISO-BOB project funded by the U.S. Office of Naval Research (ONR) included two field campaigns involving research vessels, R/V Thomas G. Thompson (June-July 2018), R/V Sally Ride (June-July 2019), and the WC-130J Hercules aircraft (June 2018) operating in the tropical Indian Ocean. WC-130J, operated by the US Air Force (USAF) 53rd Weather Reconnaissance Squadron, is extensively used in Atlantic hurricane research^40–44^. The primary objectives of MISO-BOB include unraveling the intricacies of air-sea interaction during the ‘active’ and ‘break’ phases of monsoon akin to MISO events, characterization of outflows from convective storms, and validation of coupled models.

This article presents WC-130J aircraft observations across a tropical supercluster formed over the EIO during the MISO-BOB campaign. The supercluster was associated with a convectively coupled Kelvin Wave (‘CCKW’ henceforth). The following section provides details of aircraft and onboard instrumentation. The “Observations” section describes the observed features of the tropical supercluster and the “Summary and discussion” section summarizes observations and guidelines for future studies.

## Aircraft and instrumentation details

The WC-130J aircraft is a Lockheed Martin C-130J transport aircraft carrying a suite of instruments to measure meteorological variables (Table 1). In situ air temperature and pressure are derived from a pitot static system with the help of an onboard Digital Air Data Computer (DADC) whilst humidity is measured by the Digital Dewpoint Hygrometer. Aircraft velocity relative to air is calculated by DADC from the measurements of pitot static system, the Embedded Global Positioning System (GPS)/ inertial navigation system (INS) or EGI gives the aircraft velocity relative to the ground; the difference between the two velocities gives the air velocity. In addition, the Airborne Vertical Atmospheric Profiling System (AVAPS)^45^ collects data from the dropsondes released at the desired locations to provide vertical profiles of the atmosphere. These dropsondes record air temperature, humidity, pressure, and horizontal winds through a Vaisala RS904 sensor unit. Aspen V3.4.6 software is used for processing and quality control of the sounding data from dropsondes. This software is provided by the Earth Observing Laboratory (EOL), University Corporation for Atmospheric Research (UCAR). The Stepped Frequency Microwave Radiometer (SFMR), a remote sensing instrument, gives the surface wind speeds by sensing the sea surface roughness^46^. Our comparison between the 10-m wind speed from the dropsondes and SFMR showed that the SFMR is not accurate when the surface wind speed is less than $10\,\,\hbox {ms}^{-1}$. A similar conclusion was drawn by Uhlhorn et al.^46^ after analyzing SFMR data from the 2005 Atlantic hurricane season. During the MISO-BOB observation period, the surface wind speed was mostly below $10\,\,\hbox {ms}^{-1}$, hence observations from SFMR are not included in this study.

**Table 1:** List of the instruments onboard the WC-130J aircraft.

Instruments	Variables (Range)
Embedded Global Positioning System/INS	Latitude (− 90–90^∘^), Longitude (− 180–180 ^∘^), Altitude (− 305–15240 m)
Pitot Static System - Digital Air Data Computer	Pressure (100-1050 mb), Air temperature (− 70–40 ^∘^C), Winds (0–300 kts)
Digital Dewpoint Hygrometer	Dewpoint temperature (− 75–50 ^∘^C)
Stepped Frequency Microwave Radiometer	Surface winds (0–583 kts), Rainfall rate (0–300 mm)

### Other datasets used

The bimodal index proposed by Kikuchi^47^ is used here to characterize the state of ISO as it captures the eastward as well as the northward component of ISO. IR (10.2–11.2 $\mu$m) brightness temperature images from the INSAT-3D^48^ satellite are used to identify the regions of deep convection. INSAT-3D images are provided by the Space Application Centre (SAC), Indian Space Research Organization (ISRO). European Centre for Medium-Range Weather Forecasts (ECMWF) fifth generation reanalysis (ERA5)^49^ dataset is used to depict the large-scale meteorological environment. ERA5 is available at 37 pressure levels and 31 km horizontal resolution at hourly intervals. Version 2 of total precipitable water (TPW) over the ocean from the Cooperative Institute for Meteorological Satellite Studies (CIMSS) Morphed Integrated Microwave Imagery at CIMSS TPW product (MIMIC-TPW2) is used to gauge the total water vapor content in an atmospheric column^50^. MIMIC-TPW2 is available on a global $0.25^{\circ }$ $\times$ $0.25^{\circ }$ grid at 1-h intervals and has an accuracy of 0.5-2 mm. The MIMIC-TPW2 product is supported by the Joint Polar Satellite System (JPSS) Risk Reduction Program and ONR. Daily Optimum Interpolation Sea Surface Temperature (OISST)^51^ is used for the SST field. OISST is provided by the National Atmospheric and Oceanic Administration (NOAA) and has 1/4^∘^ spatial resolution. The Final Run 06 version of Integrated Multi-satellitE Retrievals for GPM (IMERG)^52^ is used to depict the rainfall field which gives the surface rainfall field over a global 0.1^∘^ $\times$ 0.1^∘^ grid at 30-minute intervals. IMERG data is provided by the National Aeronautics and Space Administration (NASA). The NOAA Interpolated Outgoing Longwave Radiation (OLR)^53^ is used for extracting the tropical convective activity on the intraseasonal time scale by bandpass filtering.

## Observations

### Atmospheric and oceanic conditions

**Figure 1:**
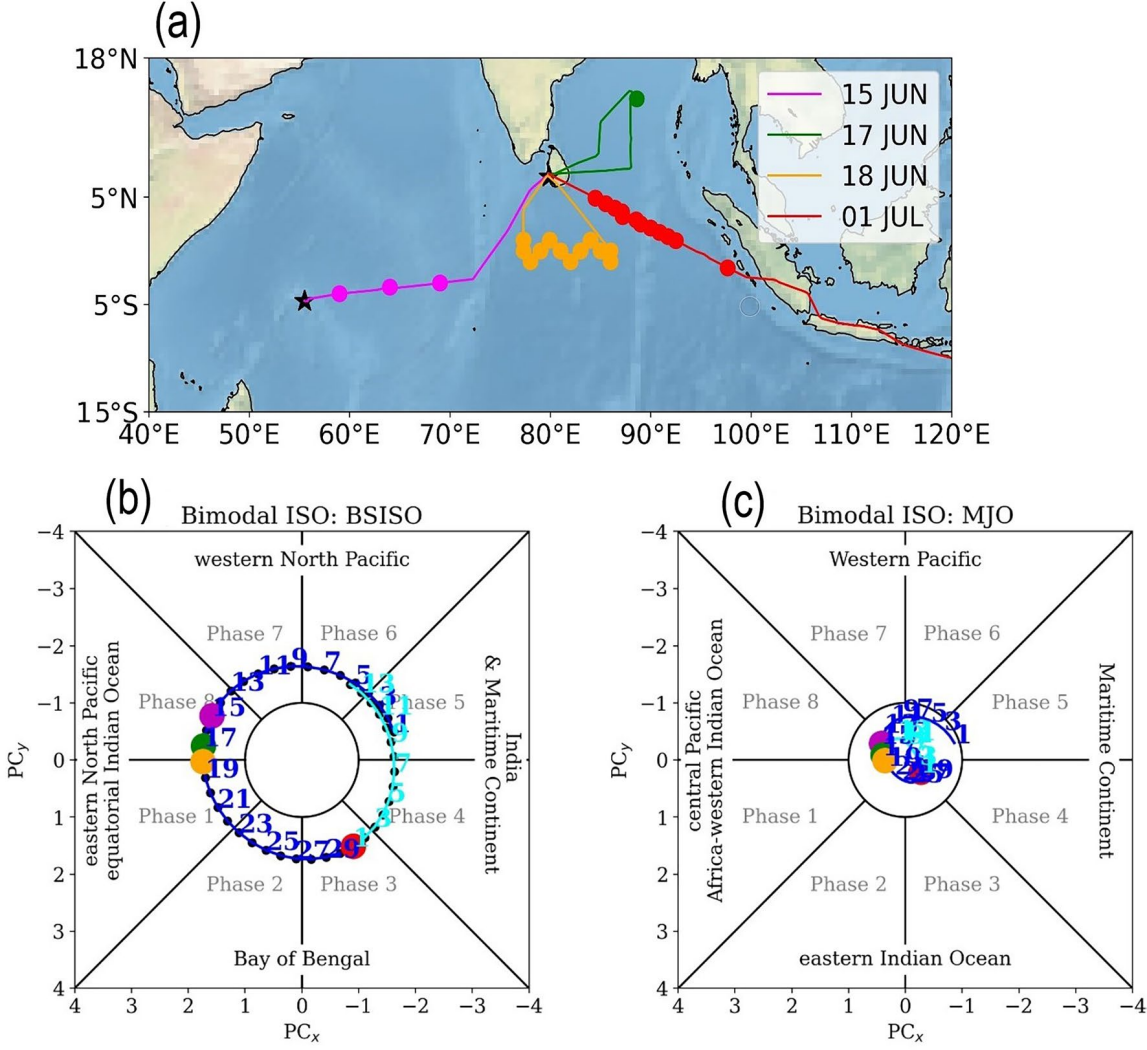
Top panel: (**a**) Tracks of WC-130J during the MISO-BOB field campaign. The dots in the tracks show the locations of dropsonde releases, and the black star symbols show the locations of Seychelles (4.68^∘^S,55.49^∘^E) and Colombo (6.92^∘^N,79.86^∘^E), Sri Lanka. Bottom panel: Evolution of (**b**) Boreal Summer Intraseasonal Oscillation (BSISO) and (**c**) Madden-Julian Oscillation (MJO) indices from Kikuchi (2020)^47^ during the WC-130J observation period (blue - June; cyan - July). The large color dots correspond to the dates of the aircraft flights shown in (**a**). The ISO is active when $\sqrt{PC_{x}^{2} + PC_{y}^{2}} > 1$ i.e. when the indices lie outside the circle in the respective plot.

Figure 1a shows WC-130J flight tracks during the MISO-BOB field campaign. Colombo (6.92^∘^N,79.86^∘^E), Sri Lanka, was the operating base for the aircraft during the campaign. It arrived at Colombo on 15 June 2018 from Biloxi, Mississippi, USA via Seychelles (4.68^∘^S,55.49^∘^E), and conducted flights on 17, 18 June, and 01 July. Technical issues in the aircraft prevented flights between 19–30 June. Thereafter, the aircraft had to return to the USA for the Atlantic hurricane season. The average cruising altitude of all flights in the MISO-BOB campaign was around 8000 m. Kikuchi (2020)^47^ bimodal ISO indices show that a strong BSISO (and a weak MJO) signal was present over the EIO during 15-21 June (Fig. 1b,c) . This suggests that the eastward-moving ISO also had a significant northward component. Around 25 June, the BSISO arrived over the Bay of Bengal and remained active there until 03 July.

**Figure 2:**
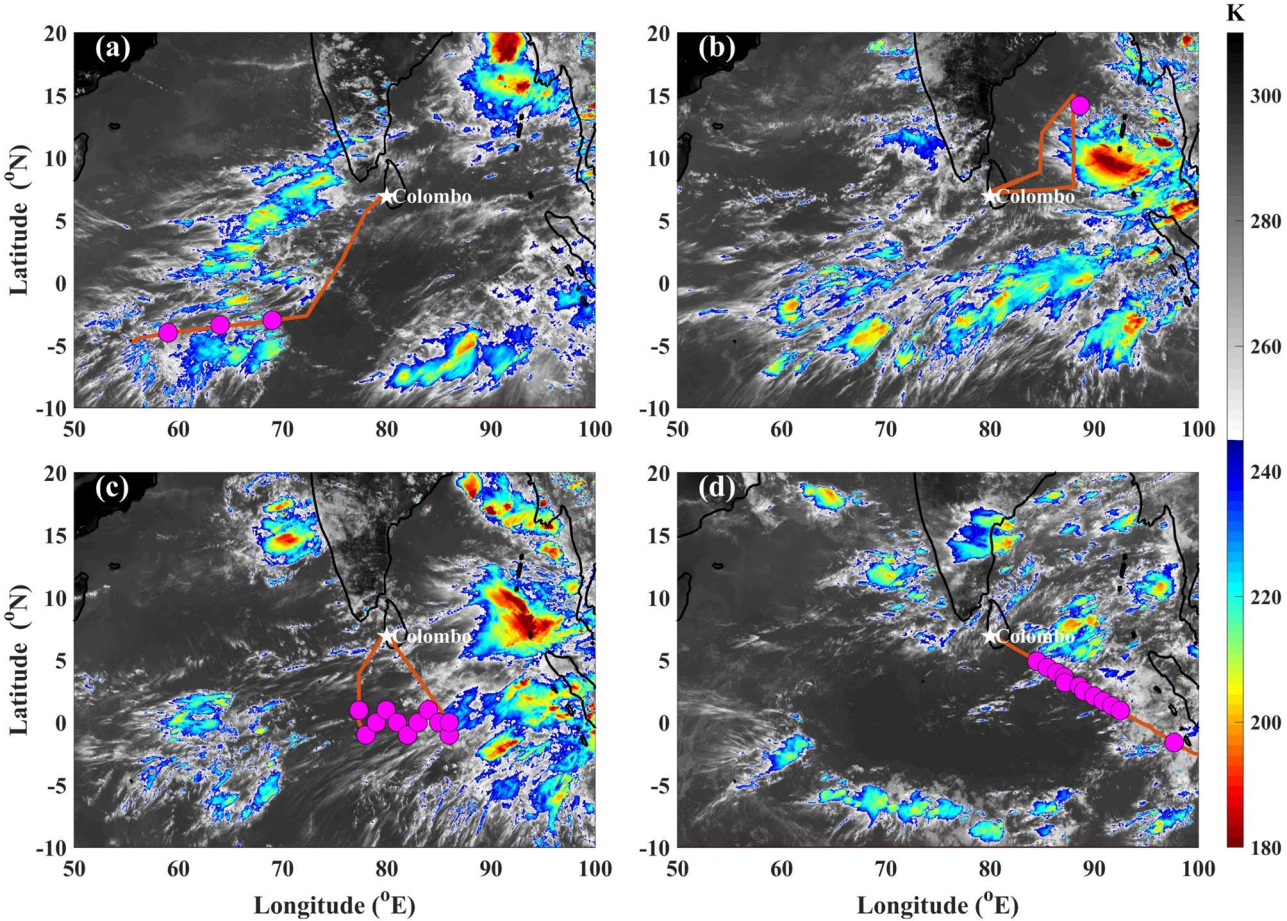
IR brightness temperature images from INSAT-3D satellite: (**a**) 1100 UTC 15 June, (**b**) 0800 UTC 17 June, (**c**) 0900 UTC 18 June, (**d**) 0400 UTC 01 July 2018. Flight tracks (orange lines) and dropsonde locations (magenta circles) on the respective days are also overlaid. The dropsonde on 17 June was over the R/V Thomas G. Thompson.

The aircraft flew through a tropical supercluster, taking a west-to-east track over the western EIO on 15 June (Fig. 2a). If a large-scale ($\sim$ O($1000\,\hbox {km}$)) organization of cloud clusters in the tropics persists for at least two days, it can be called a ‘tropical supercluster’^54,55^. The first dropsonde ( D_rear_) was dropped at the rear end of the supercluster, second in the middle (D_mid_), and third at its front end (D_frnt_). On 17 June, the supercluster moved over the central EIO (Fig. 2b), whence the aircraft flew over the Bay of Bengal and dropped a dropsonde over the R/V Thomas G. Thompson, which was stationed there. The aircraft also made a spiral descent at this location (known as a ‘calibration run’) to validate the onboard sensors (Fig. S1 in the supplemental material). On 18 June, convection moved over the eastern EIO (Fig. 2c); the aircraft flew over the central EIO and dropped several dropsondes traveling in a zigzag path about the equator in order to get the cross-section of the equatorial atmosphere. During its return to its base in the USA, via Darwin, Australia, WC-130J released dropsondes over the eastern EIO (Fig. 2d).

**Figure 3:**
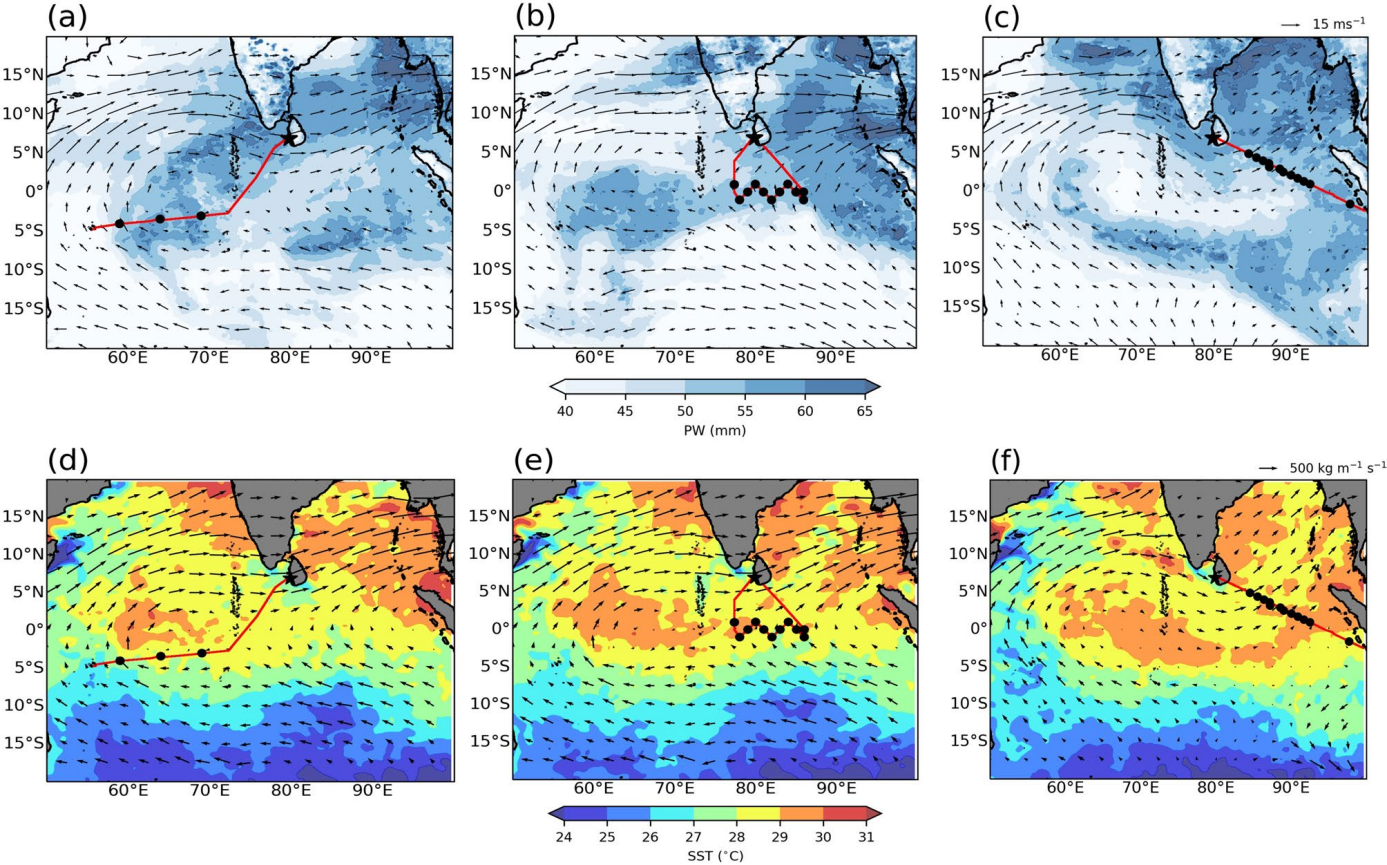
Daily means of total precipitable water (TPW) from MIMIC-TPW (version 2) and 850 hPa winds from ERA5 on (**a**) 15 June, (**b**) 18 June, (**c**) 01 July; daily means of SST from OISST and vertically integrated horizontal water vapor flux from ERA5 on (**d**) 15 June, (**e**) 18 June, (**f**) 01 July. Flight tracks on the respective days are also overlaid.

During 15–18 June, due to the active BSISO conditions over the EIO, high TPW values were seen over this region (Fig. 3a,b). By 01 July, the ISO moved over the Bay of Bengal; hence the atmosphere over EIO has dried, and high TPW values are seen over the Bay (Fig. 3c). Figure 3d–f show daily mean vertically integrated horizontal water vapor flux vectors and SST fields. The vertically integrated water vapor flux vectors are aligned with the 850 hPa winds indicating the importance of boundary layer dynamics in transporting water vapor. Two prominent bands of high SST values are seen over the region, one over the Arabian Sea and Bay of Bengal, and the other over EIO. During the monsoon season, a planetary scale convective band due to the inter-tropical convergence zone (ITCZ) alternately forms over these two high SST zones leading to MISO^1^. The SST values at the dropsonde locations are around 28–29 ^∘^C.

**Figure 4:**
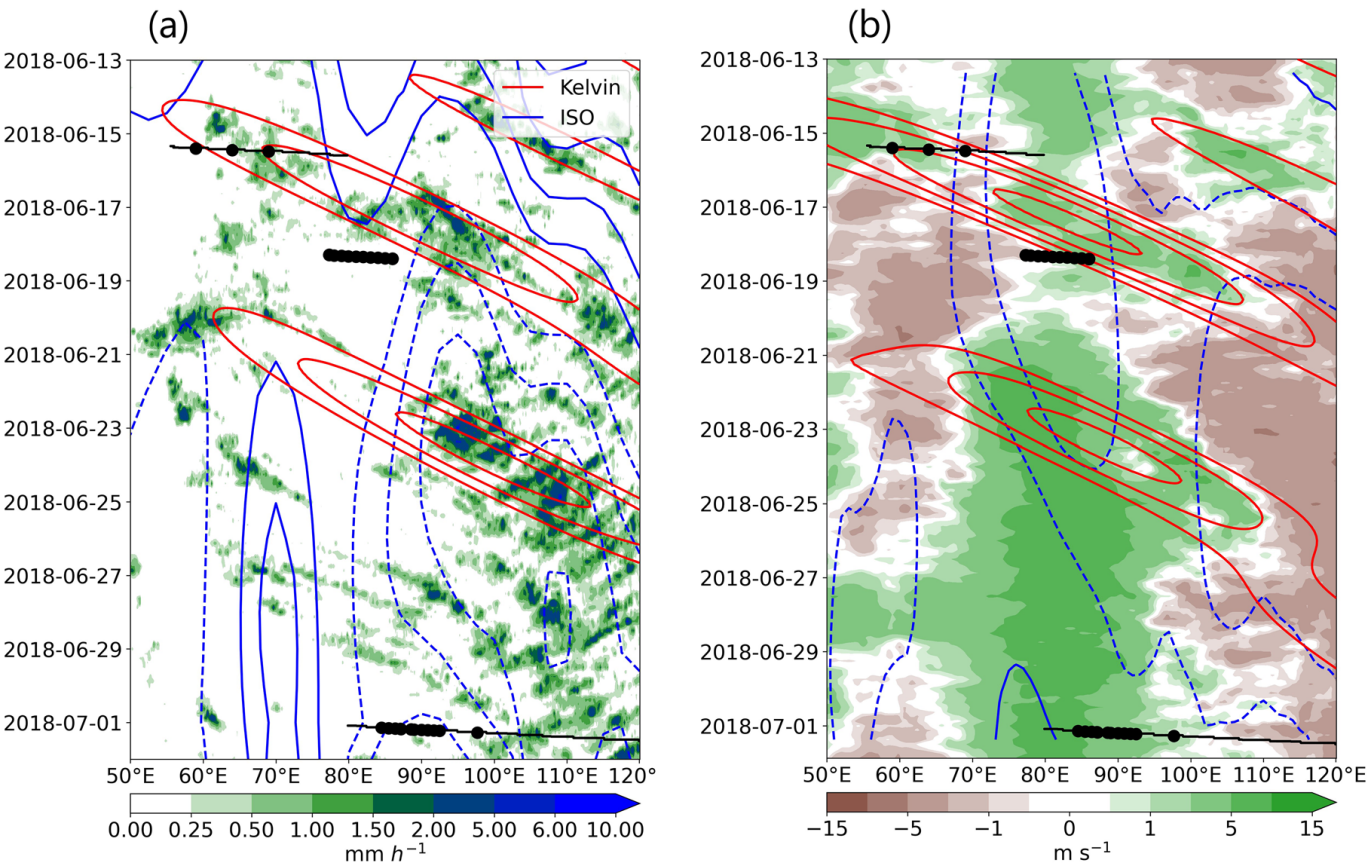
(**a**) Hovmöller diagram of 5^∘^S-5^∘^N averaged IMERG rainfall. The red (blue) contours show rainfall (OLR) anomalies with 0.1, 0.2, 0.3 mm hr^-1^ (5, 10, 15, 22 W m^-2^) values over the same latitudinal band filtered for CCKW (30-60 day ISO). The dashed contours show negative anomalies. (**b**) Same as (**a**) but for the ERA5 850 hPa zonal winds. The red contours show wind anomalies (0.5, 1, 1.5, 2, and 3 m $s^{-1}$) filtered for CCKW and the blue contours show for 30–60 day ISO (at 1 m s^-1^ intervals). The dashed contours show positive anomalies. The black lines in (**a**) and (**b**) show flight tracks and the dots show dropsonde locations.

Figure 4a shows Hovmöller diagram of 5^∘^S-5^∘^N averaged rainfall from IMERG data. The 15 June supercluster was associated with a CCKW event, referred to as KW1 henceforth, whose initiation over the western EIO coincides with the organization of the supercluster. KW1 propagates eastward with a speed of around $12\,\hbox {ms}^{-1}$ over EIO. Convection remains active over the western EIO even after the passage of KW1 and spurs another CCKW (KW2) on 20 June. KW2 leads to substantial rainfall and active ISO phase over the Maritime continent region (90–120^∘^E). Thus, the warm water patch over western EIO (60–70^∘^E) appears to be a breeding ground or a refueling station for tropical planetary waves. The KW1 and KW2 events are associated with enhanced westerly winds (Fig. 4b). Note that the enhancement of westerly winds and rainfall are closely related. The WC-130J aircraft flew across KW1 on 15 June and in its wake on 18 June.

### Dropsonde observations

#### Conditional instability

**Figure 5:**
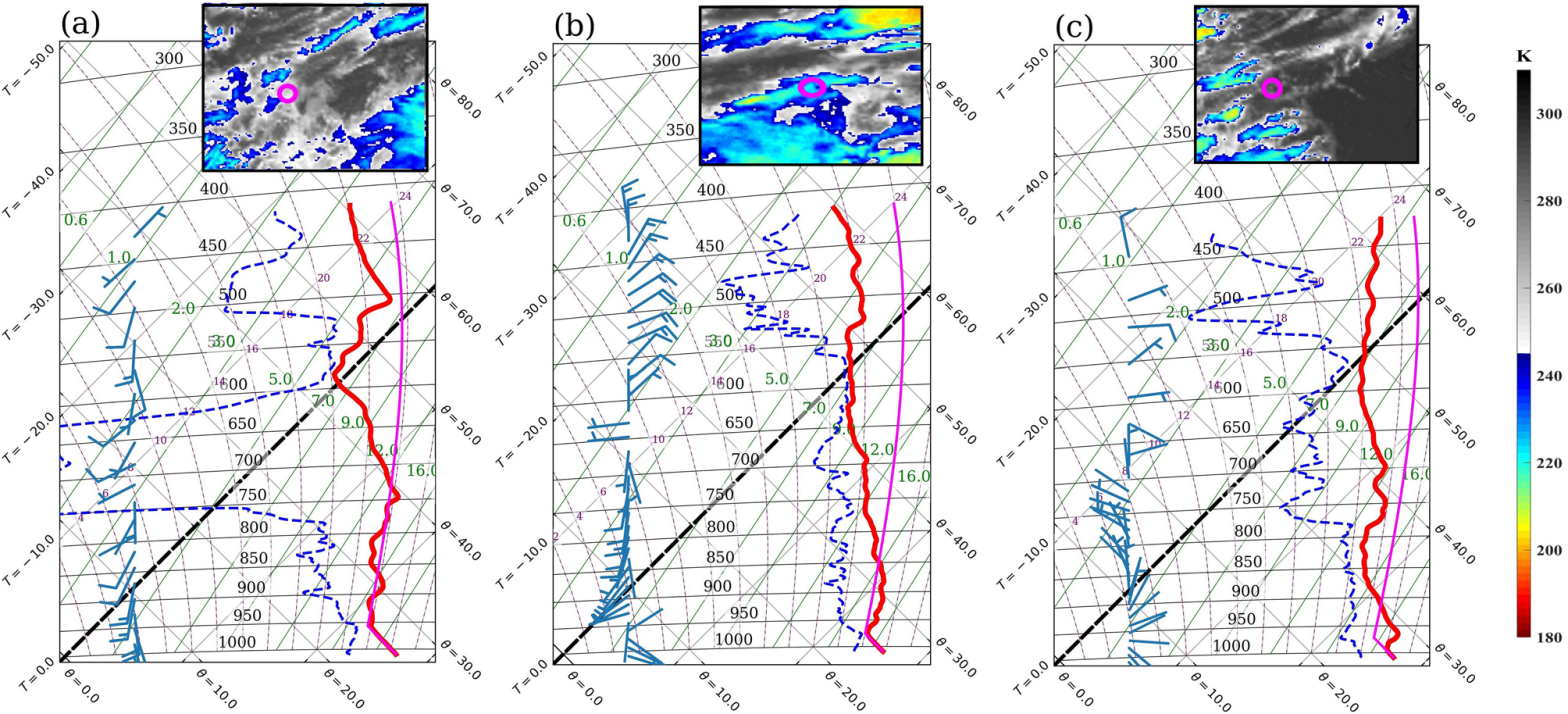
Sounding profiles on tephigram: (**a**) $D_{rear}$, (**b**) $D_{mid}$, and (**c**) $D_{frnt}$; red line shows the dry bulb temperature, dashed blue line the dewpoint temperature, magenta line the calculated temperature of parcel uplifted (adiabatically below LCL and pseudoadiabatically above LCL) from the surface, and barbs show wind vectors. The inset figures show the locations of dropsondes on INSAT-3D IR brightness temperature images around the respective times: (**a**) 0930 UTC, (**b**) 1030 UTC, and (**c**) 1130 UTC 15 June 2018.

Figure 5 shows soundings from the three dropsondes released on 15 June. Table 2 gives the details of these soundings. D_rear_ (Fig. 5a) shows dry air intrusion at the middle levels (750-600 hPa). Although the lifting condensation level (LCL) in D_rear_ is at $500\,\hbox {m}$ elevation, the level of free convection (LFC) above which the conditional instability exists is at $2805\,\hbox {m}$. The level of neutral buoyancy (LNB), the theoretical upper limit of deep convection, lies well above 400 hPa, and thus could not be captured by any of the dropsondes, and so is the amount of instability. LFC for D_mid_ lies at $1522\,\hbox {m}$, and the LCL lies at $310\,\hbox {m}$ (Fig. 5b). LCL in D_frnt_ is also at a similar level (Fig. 5c). However, unlike D_rear_ and D_mid_, the atmosphere at LCL in D_frnt_ is far from saturation. D_frnt_ has the lowest LFC at 897 m and the greatest instability among the three soundings. The convective inhibition (CIN) values, based on mixed layer temperatures, for D_rear_, D_mid_, and D_frnt_ are − 90 j/kg, − 73 j/kg, and − 25 j/kg, respectively. CIN gives the amount of kinetic energy required for the parcels to reach LFC. Thus, the lowest values of LFC and CIN and high instability suggest that D_frnt_ has the most favorable conditions for ensuing deep convection. The presence of conditional instability is a necessary but not sufficient condition for developing deep convection. An uplifting mechanism is required for the release of this instability, and identifying this mechanism is crucial for convective parameterization^56^.

**Table 2:** Details of the dropsondes released on 15 June 2018.

Dropsonde	Time (UTC)	Location (lat,long)	LCL (m)	LFC (m)	Mixed layer height (m)	CIN (j/kg)
$D_{rear}$	09:39	− 4.0, 59.0	500	2805	515	− 90
$D_{mid}$	10:39	− 3.4, 64.0	310	1522	336	− 73
$D_{frnt}$	11:39	− 3.0, 69.0	338	897	188	− 25

Mixed layer height is based on the virtual potential temperature ($\theta _{v}$) profile (Fig. 8d).

*LCL* Lifting condensation level,* LFC* Level of free convection, *CIN* Convective inhibition.

#### Divergence profile

**Figure 6:**
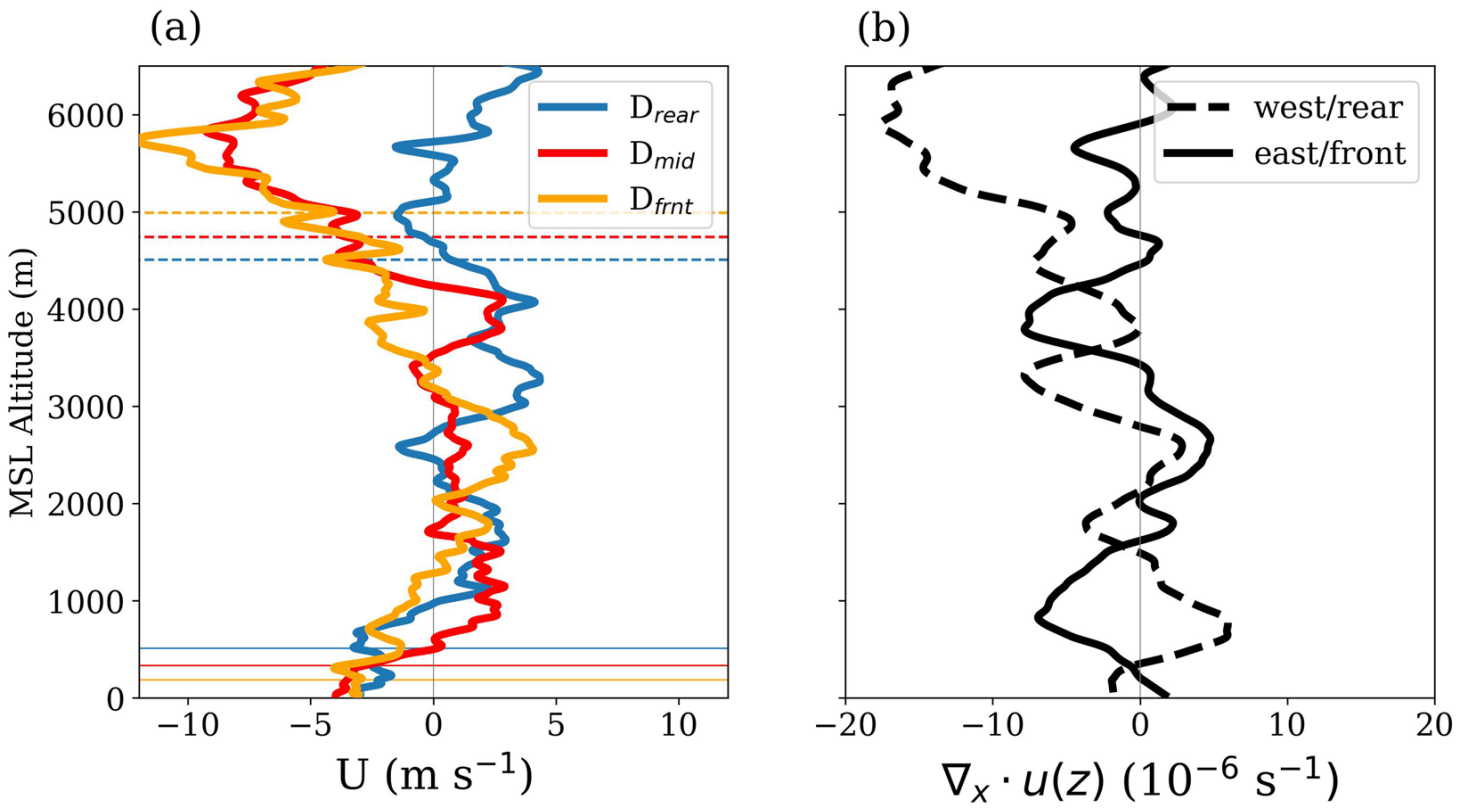
Profile of (**a**) zonal wind velocity, (**b**) zonal divergence. The dashed lines in (**a**) show the elevations of 0^∘^ C and the thin solid lines show mixed layer heights in the respective soundings.

CCKW induces anomalies in the zonal winds leading to convergence to the east and divergence to the west^57^. Our 15 June dropsonde observations show that above the boundary layer, winds at the rear mostly have a westerly component, and at the front, they have mostly an easterly component (Fig. 6a). Zonal winds in the middle of the supercluster are westerly in the lower troposphere, hereafter referred to as low-level westerlies (LLW), and easterly in the upper troposphere, i.e., the winds show a baroclinic structure. The transition from easterly to westerly occurs below the melting level (0^∘^C). Thus, this transition seems to be caused by the downburst of the cold air formed by the melting of ice. Similar observations were reported by Chen at al.^35^ during DYNAMO field campaign over EIO.

These zonal wind profiles in the supercluster also resemble the vertical structure of CCKW shown by Kiladis et al.^57^ (Figure 8 in that article) by compositing the soundings at the Marshall Islands in the central Pacific. This resemblance supports the notion of ‘self-similarity’^57^ or the ‘building block’ hypothesis^58^ wherein the life cycles of individual mesoscale convective systems (MCSs) are aliased over large scales through linear superposition. The near-surface flow has an easterly component in all three soundings. The easterly flow at the middle and rear is due to the cold pool formed by the descending cold air from the melting and evaporation of hydrometeors. The structure of this cold pool is discussed in detail later in this section.

Figure 6b shows profiles of zonal divergence at the western and eastern halves calculated using the 15 June dropsonde data in the following manner:

1 $$\begin{aligned} \nabla _{x} \cdot \mathbf {{u(z)}_{west}} = \frac{ \Delta u(z)_{mid-rear}}{\Delta x(z)_{mid-rear}} \quad \text {and} \quad \nabla _{x} \cdot \mathbf {{u(z)}_{east}} = \frac{ \Delta u(z)_{frnt-mid}}{\Delta x(z)_{frnt-mid}} \end{aligned}$$

where $\Delta$ $u$ is the difference in the zonal winds and $\Delta$ $x$ is the zonal distance between the two sounding points at an elevation *z*. The eastern half of the supercluster has low-level zonal convergence below 2000 m, and the western half has zonal divergence causing an asymmetry between the forward and rear halves. Horizontal convergence in the boundary layer leads to ascending air motion at its top. However, since these profiles show only the zonal component of the divergence, we refrain from establishing any direct connection with the ascending motion. Nevertheless, Fig. 5b shows that winds at this level are mainly zonal within the supercluster. The LFC in $D_{frnt}$ is at 896 m (Fig. 5c), and the zonal convergence in the eastern half peaks around 1000 m elevation. This suggests that the rising motion induced by the zonal convergence can trigger new convective cells. Note that the convergence at this level is due to LLW and not due to the cold pool which is much shallower than LFC in the eastern half (shown later in the ‘Cold pool’ subsection). Above 4000 m, the western half of the supercluster has a converging profile, while the divergence profile at the eastern half has no clear preference.

**Figure 7:**
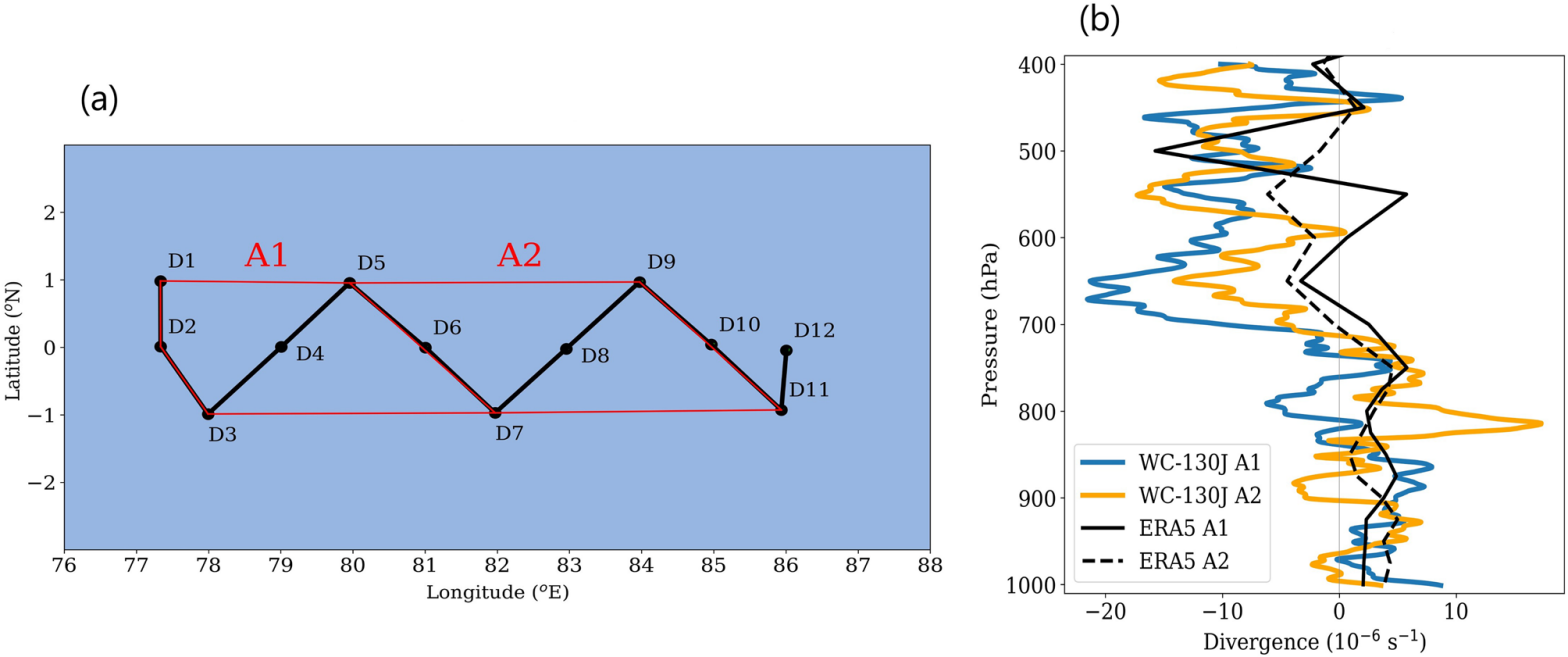
(**a**) Black lines show flight track on 18 June, dots show the locations of dropsondes, red lines show hypothetical polygons A1 and A2 used for calculating divergence profile. (**b**) Divergence profiles from dropsondes and ERA5 data over the polygons A1 and A2 shown in (**a**).

A pertinent doubt regarding the accuracy of the divergence profile calculated from the soundings separated by a few hundred kilometers can be raised. Furthermore, in the case of ship arrays, radiosondes are released simultaneously from the corners of polygon^59,60^, however, the dropsondes are released with some time lag from a single aircraft. Nevertheless, we hypothesize that large-scale flow of the tropical atmosphere evolves at a much longer time scale ($\sim$ days), if not disturbed by an MCS, compared to the time scale of aircraft measurements^36^ ($\sim$ hours, with a cruising speed $\sim$ 500 km h^-1^), hence the synoptic divergence calculated using the dropsonde data has small errors. Validation of this hypothesis is crucial for the planning of future aircraft campaigns. The flight track and the dropsonde pattern of 18 June are ideal for this task (Fig. 7a). It was selected to enable calculating the total divergence profile of the atmosphere, much similar to those reported in previous field campaigns using ship-based soundings^32,59,60^. These profiles are vital for understanding atmospheric convective heating^33^ and air-sea interactions^61^. The 18 June flight track spans the wake of KW1. Here, the divergence profile is calculated over two hypothetical polygons A1 and A2, delineated by the red lines in Fig. 7a, by using dropsonde data and the Gauss divergence theorem:

2 $$\begin{aligned} \left[ {\nabla \cdot \vec {V}_{H}(z)} \right] _{A} = \frac{1}{A} \Bigl [ \sum \left[ |\vec {v_{ni}}(z)|r_{i}(z) \right] _{in} - \sum \left[ |\vec {v_{ni}}(z)|r_{i}(z) \right] _{out} \Bigr ] \end{aligned}$$

where, $\left[ {\nabla \cdot \vec {V}_{H}(z)} \right] _{A}$ is the mean horizontal divergence at level *z* over polygon with area A, $\vec {v_{ni}}(z)$ the mean velocity vector at *z* normal to side *i* of which the length is $r_{i}$. The suffixes ‘*in*’ and ‘*out*’ refer to incoming and outgoing flow, respectively. Note that A1 and A2 are selected such that the aircraft takes about an hour to traverse each area. $\vec {v_{ni}}(z)$ is approximated as the mean of the velocity components normal to side *i* as measured by the dropsondes at the two vertices of that side. Figure 7b shows the divergence profiles over A1 and A2 derived by substituting the dropsonde velocities in Eq. (2). Mean divergence profiles over these areas from ERA5 during the flight hours are also plotted. It shows that the divergence profiles derived from dropsonde data are consistent with those from ERA5, at least with regard to trends. These profiles show divergence in the lower and convergence in the upper troposphere, in agreement with the dynamics of CCKW wakes^57^.

#### Cold pool

**Figure 8:**
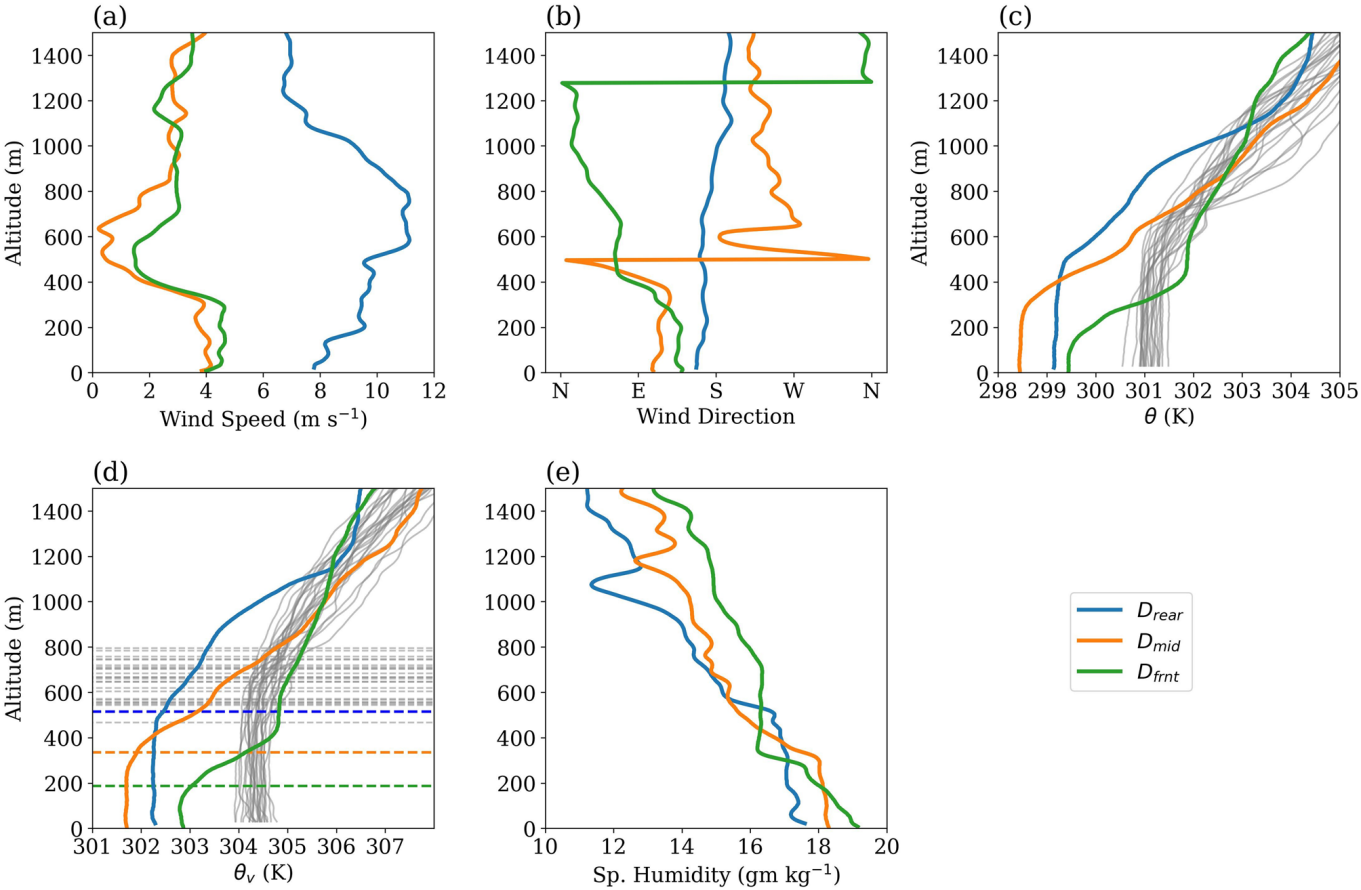
(**a**) Wind speed, (**b**) wind direction, (**c**) $\theta$, (**d**) $\theta _{v}$, and (**e**) specific humidity profiles near the surface from the 15 June dropsondes. The solid grey lines in (**c**), (**d**) show $\theta$ and $\theta _{v}$ profiles, respectively, from 18 June and 01 July dropsondes. The horizontal dashed lines in (**d**) show mixed layer heights based on $\theta _{v}$ profiles.

Figure 8a–e show the profiles of wind speed, wind direction, potential temperature ($\theta$), virtual potential temperature ($\theta _{v}$), and specific humidity (*q*), respectively, near the surface from D_rear_, D_mid_, and D_frnt_. $\theta$ profiles from the soundings on 18 June and 01 July are also shown in light grey color in the background of Fig. 8c. Surface temperatures in 18 June and 01 July soundings are around $301\,\hbox {K}$, roughly equal to the local SST values (Fig. 3e,f). These dropsondes were released in fairly clear sky locations (Fig. 2c,d) and signs of the cold pool are not seen in their profiles. On 15 June, the rear end of the supercluster is affected by a strong cross-equatorial southerly monsoonal jet (Fig. 3a). Maximum speed in this jet, about $10\,\hbox {ms}^{-1}$, is at 600-800 m elevation (Fig.8a). Maximum speeds in the middle and at the front end are seen at the surface; the surface flow at these locations is easterly-southeasterly (Fig. 3b) and its depth is around 300 m. Above 400 m, winds are westerly within the supercluster, whereas at the front they are northeasterly. Profiles of $\theta$ show that the air temperatures over the surface are colder than the local SST values (301-302 K) and deviate from the cloud-free profiles seen on 18 June and 01 July (Fig. 8c). This suggests that a cold pool was sampled by all three dropsondes. The cold pool is coldest ($\Delta \theta$ $\sim$ 2.5 K) in the middle, deepest at the rear (depth $\sim$ 1000 m), and shallowest (depth $\sim$ 300 m) at the front. Table 2 shows the mixed layer heights based on $\theta _{v}$ profiles in these soundings (Fig. 8d). The high degree of conditional instability at the front (Fig. 5c) suggests that the cold pool is advected here from elsewhere. The specific humidity in the cold pool is highest at the front and lowest at the rear (Fig. 8e). The deepening of the cold pool at the lee suggests vertical entrainment of air masses into the upper levels.

The theoretical speed of the cold pool, assuming a density current type of flow, can be given by

3 $$\begin{aligned} V_{c} = \kappa \sqrt{\left( g\frac{\theta _{v}'}{\theta _{v}}\right) h} \end{aligned}$$

where g is the gravitational acceleration, $\theta _{v}$ the environmental virtual potential temperature, $\theta _{v}'$ the difference in the environmental and cold pool virtual potential temperatures, *h* the depth of the flow and $\kappa$ is a constant with value 0.78^62^. The mixed layer of the cold pool at the surface has the following values in $D_{mid}$: $\theta '$ = 2.5 K, *h* = $300\,\hbox {m}$. Assuming $\theta _{v}$ = $304\,\hbox {K}$ (Fig. 8d), the speed of this cold pool from Eq. (3) is $4\,\hbox {ms}^{-1}$. Figure 8a shows that the observed speed of the cold pool in the middle of the supercluster matches this theoretical speed. As the environmental easterly flow ahead of the supercluster is roughly of the same magnitude, we expect a stagnation of the cold pool at the front end of the supercluster. This cold pool can uplift the warm air parcels advected from the east above LCL (at 310 m) to form the cloud base. However, there is not enough evidence to suggest that it can trigger deep convection as the LFC lies at a much higher level ($897\,\hbox {m}$) at the front end. The rear and front ends of the cold pool can be affected by large-scale/background wind flow, and determining the speed of the cold pool at these ends is difficult without a detailed knowledge of the background flow.

### Flight level observations

**Figure 9:**
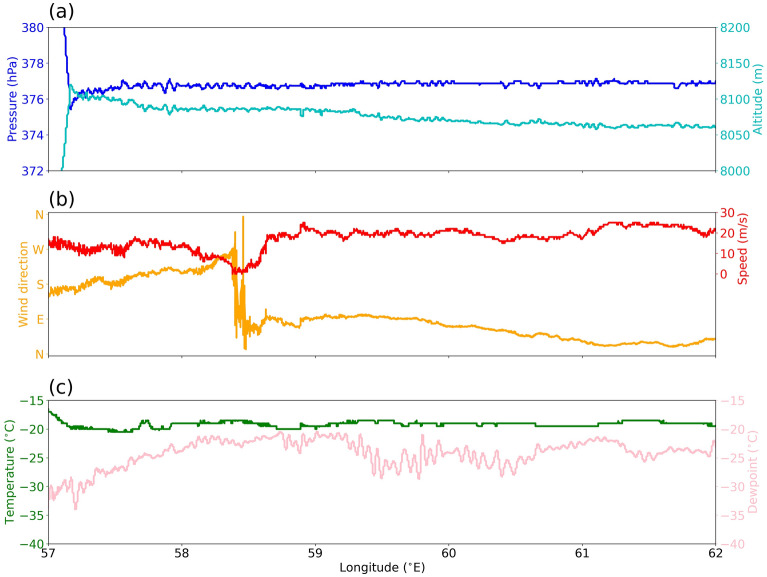
Flight level data from onboard instruments on the 15 June: (**a**) altitude and pressure, (**b**) wind speed and direction, (**c**) air temperature and dewpoint.

After taking off from the Seychelles airport, the aircraft achieved an altitude of 8050 m around 57.5^∘^E and cruised at this altitude across the supercluster (Fig. 9a). There is an abrupt change in the wind direction from south/southwesterly to northeasterly at around 58.5 ^∘^E, within a span of just $10\hbox {-}20\,\hbox {km}$ (Fig. 9b). Wind speed drops to nearly zero at this location, suggesting a stagnation region. Pressure, temperature, and dewpoint variations across this discontinuity, however, were insignificant (Fig. 9a,c). An easterly-to-westerly shift in the upper levels is expected at the rear end of CCKW due to their characteristic east-to-west tilt^57^, however, the sudden shift observed here is uncharacteristic for large-scale features like tropical waves and is likely associated with an evolving mesoscale system^63^.

## Summary and discussion

Aircraft observations during the MISO-BOB field campaign witnessed the genesis and passage of a CCKW event during 15-18 June 2018. Even though the WC-130J aircraft did not fly the intended number of (seven) sorties during the campaign due to technical glitches, observations from the 15 June flight revealed interesting features of CCKW supercluster. Owing to the paucity of relevant observations, our understanding of the interaction between convection and large-scale waves is still limited. Therefore, we still do not have satisfactory answers to the following important questions – How these large-scale waves are triggered? To what extent their evolution is coupled with convection? How do they influence the ISO events? As far as the genesis is concerned, the warm SST patch over the western EIO seems to play an important role in organizing convection. During the 2018 and 2019 MISO-BOB field campaigns, CCKW events repeatedly started over this warm SST patch. Therefore, air-sea interaction over this warm patch may be vital for triggering CCKW. By the time the WC-130J aircraft arrived over the region, convection was well organized in the form of a supercluster and the aircraft could not capture the events that lead to the organization of convection. Nevertheless, the following important facts about the tropical supercluster were revealed:

- The observations across the supercluster showed dynamical features consistent with CCKW structure illustrating the potential for investigating scale interactions between individual convective cells and wave envelope using observations of this ilk.
- The total mass divergence profile calculated from an array of dropsondes agrees well with that from ERA5. This has implications for future field campaigns on tropical convection, especially on pertinent energy and moisture budgets; aircraft- or drone-based dropsonde releases at proper locations may be a viable option.
- The westerlies within the supercluster were observed below the melting level, probably driven by a downburst induced by ice melting.
- Cold pool beneath the supercluster had temperature perturbation of $\sim$ − 2 to $\hbox {-}3\,\hbox {K}$ and specific humidity perturbation of around $\hbox {-}2\,\hbox {gkg}^{-1}$. It spread mainly to the west of the supercluster leading to an east-west dynamic and thermodynamic asymmetry rendering the eastern side favorable and the western side unfavorable for ensuing convection.
- Triggering of new convection to the east of the supercluster was likely to be caused by the convergence between the ambient easterlies and low-level westerlies within the supercluster rather than lifting by the spreading cold pool, as the LFC was at a much higher altitude than the cold pool.

**Figure 10:**
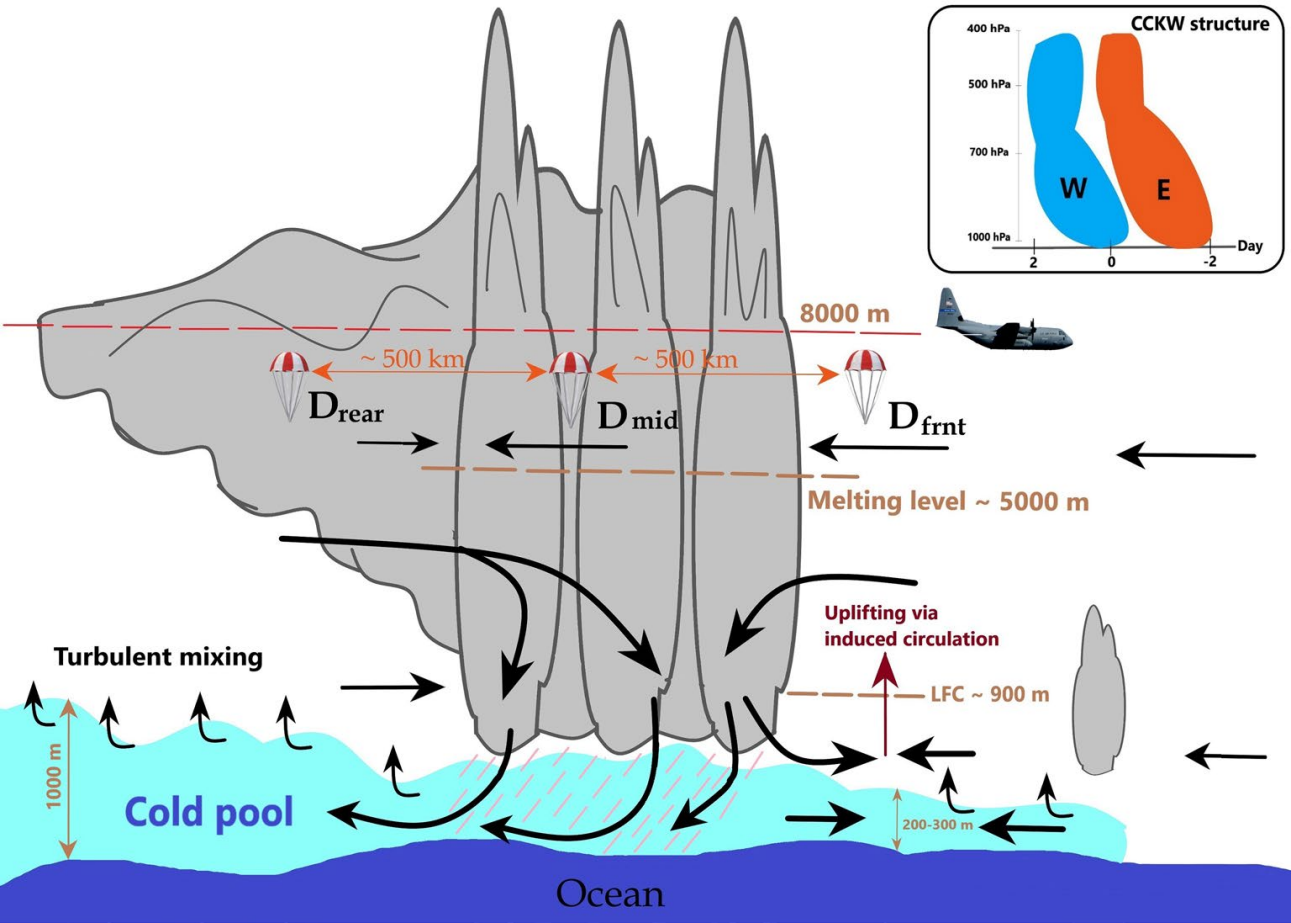
A schematic of the observations taken by the WC130J aircraft in the 15 June 2018 supercluster over the EIO. Note that the schematic is not to scale and the vertical scale is greatly exaggerated. The inset shows zonal wind structure in the CCKW given by Kiladis et al.^57^; the letters ‘W’ and ‘E’ show the regions of westerlies and easterlies, respectively, and Day 0 corresponds to the day on which IR brightness temperature is minimum. In the case of supercluster, this point coincides with the tallest convective cell. The low-level westerly ahead of the tallest cell may be driven by the cold air downburst. The clipart image of the WC130J aircraft is taken from https://www.403wg.afrc.af.mil.

These observations are depicted by a schematic in Fig. 10. Note that some of the above points may be exclusively applicable to tropical superclusters. It is possible that in a different type of convective organization, the cold pool might be deep enough to mechanically uplift unstable air parcels above LFC and trigger new convective cells, especially when the mid-troposphere is dry^64^. Nevertheless, irrespective of the form, cold pools lay the thermodynamical pathway for convective organization^65^. For the MISO-BOB program, cold pools are of particular interest from the air-sea interaction point of view. The ongoing research in this program involves analyzing observations from R/V Thomas G. Thompson and R/V Sally Ride to better understand the details of convective cold pools in the Bay of Bengal. It will be interesting to see how the cold pools influence oceanic conditions and ISO events.

### Supplementary Information

Supplementary Figure S1.

## Data Availability

The WC130J observation dataset analysed in the current study are available from the corresponding author on reasonable request. Other datasets are publicly available and their sources are mentioned in the “Other datasets used” subsection.
