# Supplementary material for: Aircraft observations in a tropical supercluster over the equatorial Indian Ocean during MISO-BOB field campaign
Source: Sci Rep. 2024 Jan 25;14:2182. doi: 10.1038/s41598-024-51527-4 (PMC10810973; doi:10.1038/s41598-024-51527-4)

Supplemental material: Aircraft Observations in a Tropical Supercluster over the Equatorial Indian Ocean during MISO-BOB Field Campaign.

Figure S1: (a) Descents of WC130J aircraft and dropsonde on 17 June 2018. The star shows location of R/V Thomas G. Thompson in the Bay of Bengal. (b) Dry bulb temperature (solid) and dewpoint (dashed) profiles from dropsonde (0757 UTC), radiosonde (0521 UTC) and WC130J onboard sensors (0757 - 0934 UTC). (c), (d) Same as (b) but show wind speed and wind direction, respectively. The aircraft descended to an altitude of 274 m above sea level.

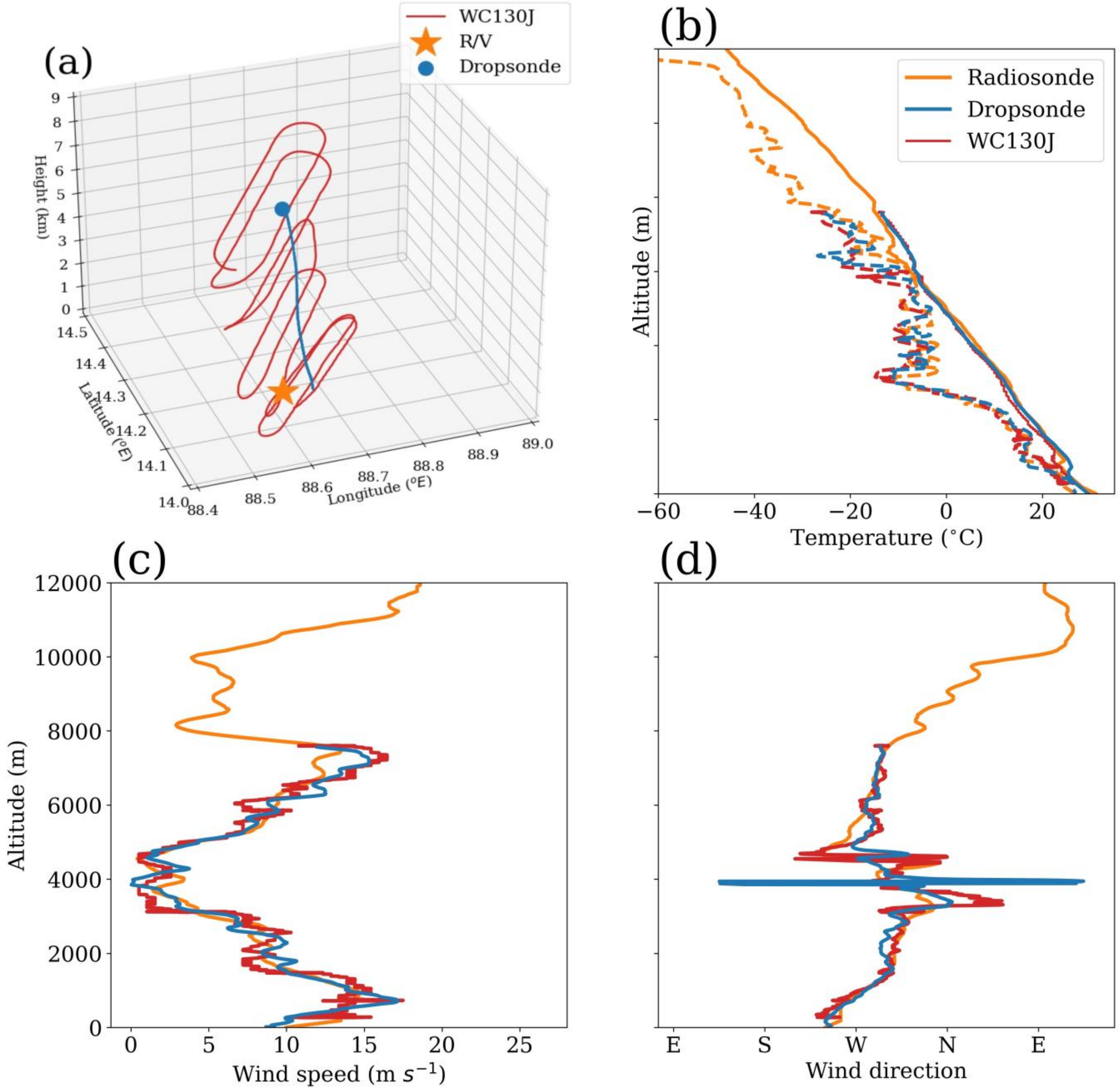

Supplement: Supplementary file 1 — Supplementary Figure S1. [file 41598_2024_51527_MOESM1_ESM.pdf]
